# Body composition in children and adolescents with non-classic congenital adrenal hyperplasia and the risk for components of metabolic syndrome: An observational study

**DOI:** 10.3389/fendo.2022.1022752

**Published:** 2022-10-20

**Authors:** Asaf Ben Simon, Avivit Brener, Anat Segev-Becker, Michal Yackobovitch-Gavan, Adi Uretzky, Anita Schachter Davidov, Angelika Alaev, Asaf Oren, Ori Eyal, Naomi Weintrob, Yael Lebenthal

**Affiliations:** ^1^ Sackler Faculty of Medicine, Tel Aviv University, Tel Aviv, Israel; ^2^ The Pediatric Endocrinology and Diabetes Unit, Dana-Dwek Children’s Hospital, Tel Aviv Sourasky Medical Center, Tel Aviv, Israel; ^3^ Department of Epidemiology and Preventive Medicine, School of Public Health, Sackler Faculty of Medicine, Tel Aviv University, Tel Aviv, Israel; ^4^ Nursing Services, Dana-Dwek Children’s Hospital, Tel Aviv Sourasky Medical Center, Tel Aviv, Israel

**Keywords:** bioelectrical impedance analysis (BIA), body composition, children and adolescents, fat percentage, metabolic syndrome (MetS) components, muscle-to-fat ratio (MFR), non-classic congenital adrenal hyperplasia (NCCAH)

## Abstract

**Background:**

Treated or untreated non-classic congenital adrenal hyperplasia (NCCAH) diagnosed in childhood could pose an increased risk of obesity and metabolic derangements in adolescence and early adulthood. We aimed to explore the interaction between muscle-to-fat ratio (MFR) and components of metabolic syndrome in pediatric subjects with NCCAH.

**Methods:**

This retrospective observational study was conducted in the Tel Aviv Medical Center from January 2018 to January 2022. The study group comprised 75 subjects (26 males) with NCCAH (61 hydrocortisone-treated [21 males] and 14 untreated [5 males]) and 134 healthy sex- and age-matched subjects (41 males) with normal puberty served as controls. Body composition was measured by bioelectrical impedance analysis (BIA) and muscle-to-fat ratio (MFR) z-scores were calculated. Stepwise linear regression models were applied to evaluate explanatory variables for MFR z-scores, blood pressure percentiles, lipid profiles, and glucose metabolism.

**Results:**

The median age [interquartile range] was 7.5 years [5.3, 8.8] at NCCAH diagnosis and 12.3 years [8.9, 15.4] at BIA. The median cumulative hydrocortisone dose was 7620 mg/m^2^ [2547, 12903]. Subjects with NCCAH had higher mean BMI z-scores and lower median MFR z-scores compared to controls [(0.47 ± 0.97 vs. -0.19 ± 1.04, *p*<0.001) and (-0.74 [-1.06, -0.14] vs.-0.37 [-0.99, 0.15], *p*=0.045), respectively]. The linear regression models dependent variables and their explanatory variables were: MFR z-score (R^2^= 0.253, *p*<0.001) - socioeconomic position index (β=0.348, *p*=0.003), birthweight z-score (β=-0.258, *p*=0.013), and duration of hydrocortisone treatment in years (β=0.048, *p*=0.023); systolic blood pressure percentile (R^2 ^= 0.166, *p*<0.001) - MFR z-score (β=-9.75, *p*<0.001); TG/HDL ratio (R^2 ^= 0.116, *p*=0.024) - MFR z-score (β=-0.300, *p*=0.024). No significant variables were found for glucose.

**Conclusions:**

Children and adolescents with NCCAH have a body composition characterized by an imbalance between muscle and fat tissues, which may place them at increased risk for early-onset cardiometabolic derangements. It is reassuring that glucocorticoid therapy aimed to alleviate androgen overproduction does not appear to adversely affect their body composition.

## Introduction

Non-classic congenital adrenal hyperplasia (NCCAH) is a group of enzymatic disorders characterized by a mild defect in cortisol biosynthesis. The most common form of NCCAH is caused by variations in the *CYP21A2*, the gene encoding for the 21-hydroxylase enzyme (1–3). Symptoms are variable and depend upon the extent and duration of postnatal hyperandrogenism. Individuals may be asymptomatic or display premature adrenarche and pubarche, accelerated linear growth with bone age advancement and compromised adult height, central precocious puberty, acne, hirsutism, menstrual disorders, and infertility (4). Glucocorticoid therapy is not always indicated but rather reserved and tailored for symptomatic cases of hyperandrogenism, in an attempt to alleviate androgen overproduction (5). The therapeutic spectrum of glucocorticoids is narrow, and the supraphysiological doses that are often needed to control the hyperandrogenism may have adverse metabolic implications, such as weight gain, increased blood pressure, hyperglycemia, and hyperlipidemia (6). Alternatively, sustained hyperandrogenism (in untreated/undertreated cases) may also affect body composition and metabolism. Chronic androgen excess has been reported in association with increased visceral adiposity and insulin resistance and their metabolic consequences (7).

There is scarce knowledge about the metabolic consequences of treatment with glucocorticoids and/or hyperandrogenism in pediatric patients with NCCAH. One early study reported an increased rate of obesity and impaired insulin sensitivity [as assessed by Homeostatic Model Assessment for Insulin Resistance (HOMA-IR)] in adults but not in children with NCCAH (8). Hyperinsulinemia and insulin insensitivity associated with hyperandrogenism were reported in untreated NCCAH women (9), and adolescents with NCCAH were found to be at a higher risk of increased artery intima-media thickness in an even earlier study (10). Contrarily, a recent study reported that patients with NCCAH diagnosed in childhood, whether treated or untreated, were not found to be at increased risk for overweight, obesity, or metabolic derangements in adolescence and early adulthood (11).

An increased rate of obesity and a sedentary lifestyle have resulted in a marked increase in the prevalence of cardiovascular disease (CVD) risk factors (hypertension, altered glucose metabolism, dyslipidemia, and abdominal obesity) in adulthood (12–17) and in adolescence (18–24). Our Pediatric Endocrine Unit implemented bioelectrical impedance analysis (BIA) of body composition in January 2018 as part of the routine assessment of patients referred for endocrine consultation (25). We subsequently reported the predictive value of muscle-to-fat ratio (MFR) z-scores in assessing CVD risk factors in youth with overweight and obesity (26). There are, however, limited data on obesity and cardiometabolic derangements among pediatric patients with NCCAH (27). In this study, we explored the interaction between body composition parameters and CVD risk factors in children and adolescents with NCCAH, with special focus upon the contributory role of hyperandrogenism and steroid therapy.

## Methods

### Study population

**Figure 1:**
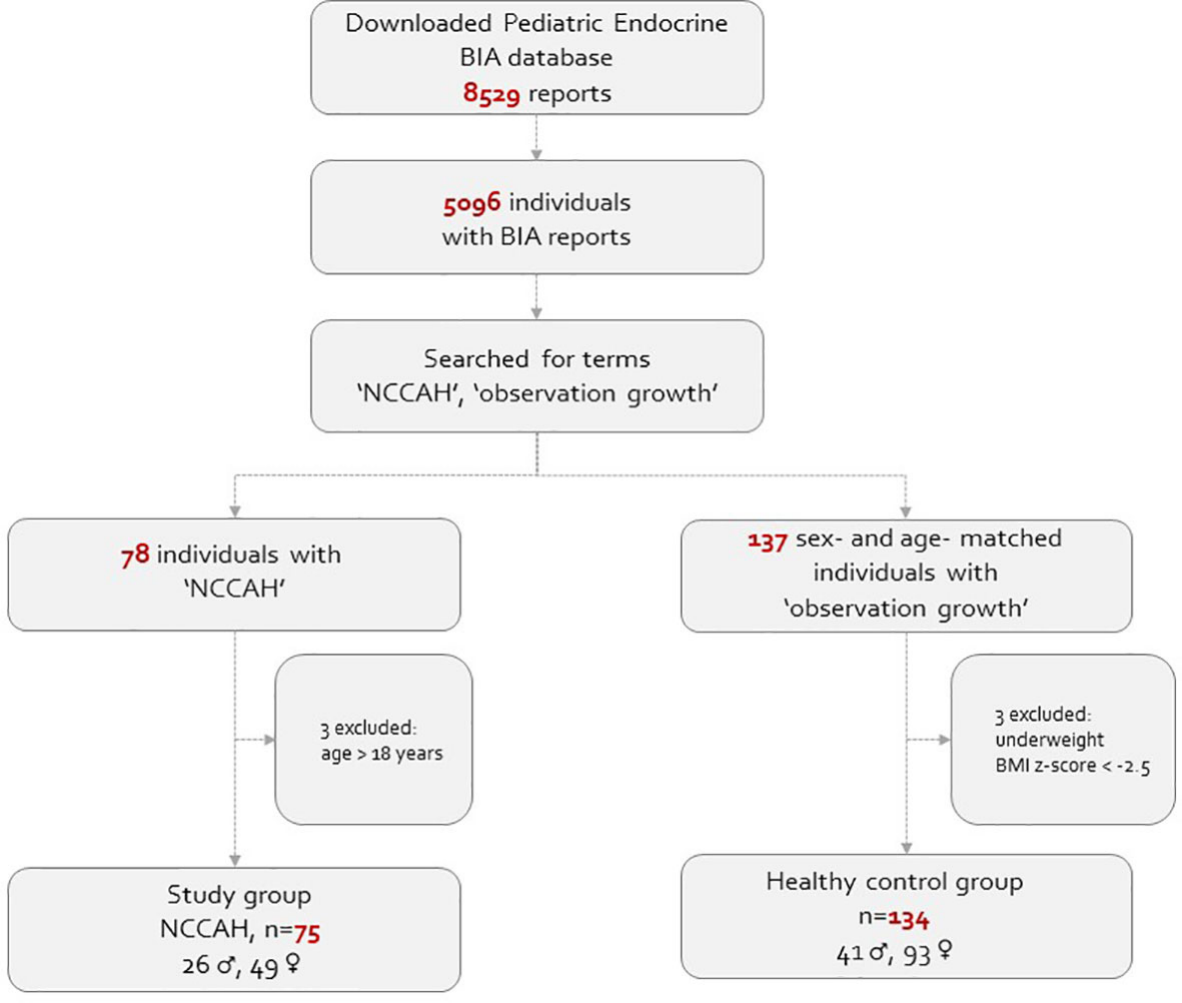
The study population flowchart.

This real-life observational study comprised of pediatric subjects (5-18 years of age) whose body composition assessment was routinely monitored at our endocrine unit in a tertiary medical center extended from January 2018 to January 2022. Both the NCCAH patient and healthy control groups were recruited from our endocrine unit. The BIA database was queried to generate a list of patients with the diagnosis of “NCCAH” and “observation of growth”. The electronic medical records of suitable children and adolescents were reviewed, and those who fulfilled the inclusion criteria were included in the analysis. The study group comprised 75 subjects (26 males) with NCCAH (61 hydrocortisone-treated and 14 untreated), and 134 healthy sex- and age- matched subjects (41 males) with normal puberty served as their controls. NCCAH was defined on the basis of an adrenocorticotropic hormone (ACTH)-stimulated 17-hydroxyprogesterone (17-OHP) serum level of >40 nmol/L according to published guidelines (5, 28). Fifty-six of the 75 individuals with NCCAH underwent molecular analysis. The healthy controls were defined as having normal stature (10^th^< height percentile< 90^th^) and normal timing of puberty and pubertal progression. Subjects with medical conditions which could lead to fluid retention (e.g., renal failure, congestive heart failure), severe underweight [body mass index (BMI) z-score ≤ -2.0], metabolic bone disease, malignancies, or genetic syndromes, or those who were using medications which could affect body composition were excluded from the study. The study population flowchart is presented in **Figure 1**.

### Clinical evaluation

The study protocol was approved by our medical center’s Institutional Review Board which waived informed parental consent. The data were handled in accordance with the principles of good clinical practice. The routine clinical evaluation of patients at endocrine referral includes a comprehensive medical interview. Female patients were questioned about their menstrual cycle (whether menses were present or absent and whether the cycle was regular or irregular), as well as their use of oral contraceptives. Anthropometric assessment of height [by means of a commercial Harpenden stadiometer (Holtain Ltd., Crosswell, United Kingdom)] and weight in light clothing (by BIA), as well as measurement of blood pressure (BP) were carried out at the first visit and repeated at each follow-up visit. Blood pressure was measured by a registered pediatric nurse who used the Welch Allyn Vital Signs Monitor VSM 300 (Welch Allyn, Inc., Beaverton, OR, USA) and chose the appropriate size cuff. The BP measurement was repeated up to three times with intervals of 5 minutes between measurements when the BP values were abnormal. The attending parent’s height was measured at first visit, and BIA weight assessment was offered as well. The height of an absent parent was provided by the available one.

The study participant’s physical examination included pubertal staging and a search for clinical signs indicative of hyperandrogenism, hypercortisolism, and insulin resistance. The decision to initiate glucocorticoids for NCCAH patients was at the discretion of the pediatric endocrinologist who was monitoring the patient. Therapy was reserved for symptomatic patients (children with premature and/or rapid progression of pubarche or bone age advancement and adolescent girls with hyperandrogenism). Titration of the glucocorticoid dose was according to a composite of growth trajectory, weight gain, clinical signs of hyperandrogenism, a hormonal profile designed to maintain 17OHP levels slightly above the upper limit of the normal, and androstenedione and testosterone levels at sex, age, and Tanner stage-appropriate levels, and bone age advancement. Gonadotropin-releasing hormone analogue (GnRHa) treatment was considered for individuals with central precocious puberty. The combination of GnRHa and growth hormone (GH) therapy was considered in individuals with compromised adult height prediction (5).

### Body composition analysis

Body composition was measured by BIA [Tanita Body-Composition Analyzer (Tanita MC-780 MA) and GMON Professional Software], which has been clinically proven to be accurate and reliable and to provide highly reproducible results (29). The BIA measurements were performed in the morning (from 8:00 AM until 11:00AM) during the routine clinic visit, preferably with the subject in a fasting state and not after strenuous physical activity. The BIA measures whole body as well as segmental analysis (trunk, upper, and lower limbs) of fat and muscle. The report includes the following data: body weight (kilograms, 0.1 kg increments), fat percentage (FATP, whole body, 0.1% increments), truncal fat percentage (TFATP, 0.1% increments), fat mass (kilograms, 0.1 kg increments), muscle mass (whole body, 0.1 kg increments), total body water percentage (TBW, 0.1% increments), and estimated basal metabolic rate (BMR, expressed in kcal, 1 kcal increments). Calculated variables included: appendicular skeletal muscle mass (ASMM = the sum of muscle mass of four limbs) and MFR [ASMM (kg)/fat mass (kg)]. The z-scores for MFR were calculated according to BIA pediatric reference curves (30).

### Biochemical analysis

The hormonal evaluation included 17-OHP and cortisol levels at referral, baseline and following intravenous administration of 0.25 mg Synacthen (Novartis, New York, NY, USA), with a 17OHP measurement at baseline and 30 and 60 min after ACTH injection (31). The test was conducted in the early follicular phase of the menstrual cycle in post-menarcheal girls. Follow-up basal androgen levels were measured every 4-6 months or earlier when dose adjustment was required. Hormonal analyses in serum were performed with commercial kits in the endocrine laboratory of our hospital. Cortisol was measured using the Coat-A-Count radioimmunoassay (Diagnostics Products Corporation, Los Angeles, CA, USA), testosterone level was determined by electrochemiluminescence (Rosh, Cobas E 601), 17OHP was measured with the direct quantitative enzyme immunoassay (DBC, Diagnostic Biochem Canada Inc), and the androstenedione level was determined by chemiluminescence using the Immulite 2000 Xpi immunoassay system (Siemens) (32).

Annual fasting measurements of glucose levels and lipid profile tests were performed as part of the routine standard of care. Serum glucose was measured with the glucose oxidase colorimetric method (Hitachi 917 automated analyzer, Roche Diagnostics, Mannheim, Germany), and serum total cholesterol (TC), triglycerides (TG), and high-density lipoprotein (HDL) levels were measured with the enzymatic colorimetric method (Hitachi 904 automated analyzer, Roche Diagnostics). Low-density lipoprotein (LDL) was calculated according to the Friedewald formula: LDL = $\text{TC}-\text{HDL}-\frac{\text{TG}}{5}$ . Fasting plasma TG concentrations of >110 mg/dL were considered elevated, and an HDL <40 mg/dL was considered low (18). Homeostatic Model Assessment of Insulin Resistance (HOMA-IR) was calculated as follows: $\frac{\text{fasting insulin IU}/\text{mL X fasting glucose mg}/\text{dL}}{405}$ (33). Levels in peri-pubertal children (<11.5 years) were compared to values in European children and categorized as elevated when they were at the ≥90^th^ percentile, while levels ≥1.9 were considered elevated in youths (34).

### Data collection

All hospital medical records are electronic, with access to the individual’s health maintenance organization laboratory data. Sociodemographic characteristics, ethnicity, perinatal history, medical conditions, medications and family history of cardiovascular disease risk factors were retrieved from the medical files. Clinical data, anthropometric measurements, vital signs, pubertal staging, and laboratory evaluation were extracted at the time points of BIA assessments.

### Definition of study variables

#### Socioeconomic position

The SEP by home address [SEP cluster and SEP index] was analyzed based on the Israel Central Bureau of Statistics’ Characterization and Classification of Statistical Areas within Municipalities and Local Councils by the Socio-Economic Level of the Population (35). Residential SEP cluster, which was based on locality of residence, was coded on a 1-10 scale and grouped into low (1–4), medium (5–7), and high (8–10) categories. The SEP index is an adjusted calculation of 14 variables that measure social and economic levels in the domains of demographics, education, standard of living, and employment, ranging from the lowest (-2.797) to the highest (+2.590).

#### Clinical characteristics

BMI was calculated as weight in kilograms divided by height in meters squared. The patient’s height, weight, and BMI values were converted to sex- and age-specific standard deviation scores (z-scores) according to the CDC 2000 growth charts (36). Weight status was defined according to BMI z-scores as follows: underweight as BMI percentile ≤5^th^ percentile (z-score ≤-1.65), overweight as BMI percentile ≥85^th^ and <95^th^ percentiles (1.04 ≤ z-score <1.65), and obesity as BMI percentile ≥95^th^ percentile (z-score ≥1.65) (37, 38).

Birth weight z-scores were calculated by PediTools Electronic Growth Chart Calculators based on the Fenton growth chart for preterm infants (39). Appropriate birth weight for gestational age (AGA) was defined as corrected birth weight z-scores between -1.645 to 1.645, small for gestational age (SGA) as birth weight z-scores <-1.645, and large for gestational age (LGA) as birth weight z-scores >1.645. The height, weight, and BMI values were converted to sex- and age-specific z-scores according to the CDC 2000 growth charts (36). SBP and DBP percentiles were calculated by means of an online age-based pediatric BP calculator (40).

Pubertal stages were graded according to Tanner scores for testicular volume in boys and for breast development in girls. Onset of puberty was defined as genitalia Tanner stage 2 with a testicular volume >3 mL in boys and appearance of breast buds in girls, with or without sexual hair. The subject was considered fully pubertal when pubertal signs corresponded to Tanner stage 5 (41, 42).

### Glucocorticoid exposure

The glucocorticoid dosage was expressed as hydrocortisone in mg per body surface area (mg/m^2^). All available daily doses of treatment divided by the corresponding body surface area were summed to obtain annual cumulative doses of hydrocortisone. Each patient’s total cumulative hydrocortisone dose was determined by compiling these annual cumulative hydrocortisone doses (43).

### Statistical analyses

The data were analyzed with the Statistical Package for the Social Sciences software version 27 (SPSS Inc., Chicago, IL). Violin plots were created using R-studio 4.2 software with the ggplot2 package. Repeated measurements of BMI and MFR z-scores and BP percentiles taken between diagnosis and the last clinic visit served for the calculation of the mean intrapersonal and SD for each parameter. All statistical tests were two-sided. The Shapiro-Wilk test was applied to assess the normality of continuous data. The data are expressed as means ± standard deviations (SDs) for normally distributed variables and median and interquartile range [IQR] for skewed distribution. Pearson’s chi-square test was performed to compare the distribution of categorical variables between the NCCAH group and the control group. An independent sample t-test or an independent sample Mann-Whitney was performed to compare between two groups (NCCAH vs healthy controls) for continuous variables with normal or skewed distribution, as appropriate. Linear regression models using the stepwise approach were applied to assess the association between body composition parameters (MFR z-score and BMI z-score) and metabolic syndrome components (BP, TG/HDL ratio, and glucose levels) in the NCCAH group. The variables entered into the models included: sex, SEP index, family history of obesity, age (at diagnosis and at BIA), perinatal characteristics (gestational age and birthweight z-scores), hydrocortisone exposure (mean dose, treatment duration, and cumulative dose) and MFR z-scores (for metabolic syndrome components). A *p* value ≤0.05 was considered significant.

## Results

The NCCAH group comprised 75 subjects (27 [34.7%] males, median age at first BIA 11.2 years [IQR 8.2, 14.7]). Fifty-six of them (74.7%) had available genetic information in their clinical files, revealing that 27 (48.2%) were homozygous for the V281L variation and 22 (39.3%) were compound heterozygous for one mild and one severe variation.

The sociodemographic characteristics of the NCCAH group and their controls are presented in **Table 1**. Ethnic distribution of the NCCAH group revealed that 46.6% were Jews of Ashkenazi origin, 15% were Sephardic Jews, 37% were mixed Ashkenazi/Sephardic Jews, and 1.4% were of mixed Jewish/non-Jewish origin. The SEP of subjects with NCCAH was above average (the median SEP cluster was 8 [IQR 7, 9], and the median SEP index was 1.278 [IQR 0.773, 1.751]), but it was significantly lower compared to the healthy control group (*p* = 0.009). There were no significant group differences in sex, age, ethnic distribution, marital status, or number of children in the family.

**Table 1:** Sociodemographic characteristics of the NCCAH group and their healthy controls.

	NCCAH	Healthy controls	*p-*value
Number	75	134	
Sex, *n (%)*			
Males	26 (34.7)	41 (30.6)	0.545
Females	49 (65.3)	93 (69.4)	
Age at first BIA assessment			
Age, years, median [IQR]	11.2 [8.2, 14.7]	11.4 [8.6, 14.6]	0.453
Ethnicity, *n (%)*			
Ashkenazi Jew	34 (46.6)	46 (34.3)	0.384
Sephardic Jew	11 (15)	25 (18.7)	
Ashkenazi/Sephardic Jew	27 (37)	60 (44.8)	
Jew/non-Jew	1 (1.4)	3 (2.2)	
Socioeconomic position (SEP)			
SEP cluster categories, *n (%)*			
Low (1-4)	2 (2.7)	4 (3)	**0.009**
Medium (5-7)	28 (37.3)	24 (18)	
High (8-10)	45 (60)	107 (79)	
Cluster, median [IQR]	8 [7, 9]	8 [8, 9]	0.060
Index, median [IQR]	1.278 [0.773, 1.751]	1.470 [1.083, 1.903]	0.072
Household, *n (%)*			
Two-parent	71 (96)	121 (94.5)	0.403
Single-parent by choice	0 (0)	2 (1.6)	
Divorced	1 (1.3)	4 (3.1)	
Widow	2 (2.7)	1 (0.8)	
Children in the family			
Number	3 [2, 3]	2 [2, 3]	0.392
Birth order	2 [1, 2]	1 [1, 2]	0.435

Data are expressed as number and (percent) or median [interquartile range]. Chi-squared tests were performed to compare categorical variables between groups, and the Mann-Whitney test was performed to compare linear variables with skewed distribution. A p-value of ≤0.05 was considered significant. Bold indicates significant. Socioeconomic position (SEP) by cluster of localities of residence ranged from 1 to 10, with 1 being the lowest rating and 10 the highest. The SEP index is an adjusted calculation of 14 variables that measure social and economic levels in the domains of demographics, education, standard of living, and employment (range from the lowest -2.797 to the highest 2.590).

Data not documented in medical records: ethnicity 2 NCCAH, SEP 2 healthy controls, household 7 (1 NCCAH, 6 healthy controls), number of children in the family 15 (8 NCCAH, 7 healthy controls).

NCCAH, non-classic congenital adrenal hyperplasia; n, number; NCCAH, non-classic congenital adrenal hyperplasia; BIA, bioelectrical impedance analysis.

The pregnancy and perinatal characteristics of the NCCAH group and their controls are presented in **Table 2**. Most of the pregnancies in the NCCAH group were singletons (89.3%), and most followed spontaneous conception (80%). Assisted reproduction was required in 20% of the pregnancies, and it consisted of pharmacological ovulation induction in 14.3% and *in vitro* fertilization in 5.7%. *In utero* exposure to gestational diabetes mellitus (GDM) was reported in 1.4% NCCAH pregnancies, exposure to steroids in 17.2%, and exposure to both levothyroxine and aspirin in 1.4%. Delivery was spontaneous and vaginal in most cases (77.1%), elective C-section (10%), and emergency C-section or vacuum extraction (12.9%). Most infants were born at term (77.8%) and appropriate for gestational age (82.9%), with a median birth weight of 3060 grams and normal median adjusted birth weight z-scores (-0.385 [IQR -0.853, 0.230]); 19.4% were born pre-term and 11.4% were born SGA. Comparative analysis of the NCCAH study group and healthy control group revealed a similar rate of spontaneous conception with a significantly different distribution of assisted forms of reproduction (*p* = 0.004), lower rates of GDM (*p* = 0.027), a different distribution of maternal medications (*p <* 0.001) and a higher proportion of preterm births (*p* = 0.035). There were no significant group differences in the number of fetuses, mode of delivery, gestational age, or and birth weight parameters.

**Table 2:** Pregnancy and perinatal characteristics of the NCCAH group and their healthy controls.

	NCCAH	Healthy controls	*p*-value
Method of conception, *n (%)*			
Spontaneous	56 (80)	103 (83.1)	**0.004**
Ovulation induction	10 (14.3)	2 (1.6)	
IUI	0 (0)	4 (3.2)	
IVF biological parents	4 (5.7)	10 (8.1)	
IVF sperm donation	0 (0)	3 (2.4)	
IVF egg donation	0 (0)	2 (1.6)	
Maternal conditions, *n (%)*			
Gestational diabetes mellitus	1 (1.4)	12 (9.7)	**0.027**
Exposure to medications, *n (%)*			
No exposure	56 (80)	114 (92)	**<0.001**
Glucocorticoids	12 (17.2)	1 (0.8)	
Levothyroxine	1 (1.4)	6 (4.8)	
Insulin	0 (0)	3 (2.4)	
Metformin	0 (0)	0 (0)	
Aspirin	1 (1.4)	0 (0)	
Fetus, *n (%)*			
Singleton	67 (89.3)	113 (91.1)	0.676
Twin	8 (10.7)	11 (8.9)	
Mode of delivery, *n (%)*			
Spontaneous vaginal	54 (77.1)	97 (79.5)	0.860
Induction	0 (0)	1 (0.8)	
Vacuum extraction	2 (2.9)	2 (1.6)	
Elective C-section	7 (10)	13 (10.7)	
Urgent C-section	7 (10)	9 (7.4)	
Gestational age (GA)			
GA, weeks	39 [38, 40]	39 [38.4, 40]	0.205
Preterm,<37 weeks	14 (19.4)	13 (10.6)	**0.035**
Term, 38-42 weeks	56 (77.8)	110 (89.4)	
Postterm, ≥ 42 weeks	2 (2.8)	0 (0)	
Birth parameters			
Birth weight, *grams*	3060 [2700, 3375]	3000 [2700, 3300]	0.930
Birth weight, *z-score*	-0.385 [-0.853,0.230]	-0.550 [-1.030, 0.130]	0.285
Birth weight categories, *n (%)*			
SGA	8 (11.4)	13 (11.5)	0.783
AGA	58 (82.9)	96 (85)	
LGA	4 (5.7)	4 (3.5)	

Data are expressed as number and (percent) or median [interquartile range]. Chi squared tests were performed to compare categorical variables between groups, and the Mann-Whitney was performed to compare linear variables with skewed distribution. A p‐value of ≤0.05 was considered significant. Bold indicates significant. Data not documented in medical records: conception method in 15 (5 NCCAH, 10 healthy controls), gestational diabetes mellitus, and medications administered during pregnancy in 15 (5 NCCAH and 10 healthy controls), number of fetuses in 10 healthy controls, mode of delivery in 17 (5 NCCAH and 12 healthy controls), gestational age in 16 (3 NCCAH and 13 healthy controls), birth weight in 25 (4 NCCAH and 21 healthy controls).

NCCAH, non-classic congenital adrenal hyperplasia; GDM, gestational diabetes mellitus; SGA, small for gestational age; AGA, appropriate for gestational age; LGA, large for gestational age.

The median age of the NCCAH study group at diagnosis was 7.5 years [IQR 5.3, 8.8], and most of them were prepubertal at diagnosis (*n* = 55, 73.3% in Tanner 1 gonadarche and *n* = 33, 44% in Tanner 1 adrenarche), with a minority in full puberty (*n* = 4, 5.3%). The reasons for endocrine referral in descending order of frequency were premature adrenarche (*n* = 37, 49.3%), a parent and/or sibling with NCCAH (*n* = 22, 29.3%), bone age advancement (*n* = 8, 10.7%), central precocious puberty (*n* = 3, 4%), short stature (*n* = 3, 4%), and hirsutism (*n* = 2, 2.7%). Anthropometric measurements (mean ± SD) at NCCAH diagnosis revealed a height z-score of 0.30 ± 1.09, a weight z-score of 0.4 ± 1.05, and a BMI z-score of 0.37 ± 1.11, with a median bone age advancement of 15 months. Most of the NCCAH group (80%, 21 boys and 40 girls) received glucocorticoid therapy starting at a median age of 7.8 years and ceasing at a median age of 13.9 years; 14 children (5 boys and 9 girls) were treatment-naïve at the time of the BIA. The median duration of therapy was 36.5 months [IQR 10.5, 68.9], and the median dose was 6.59 mg/m^2^ with a lifetime cumulative glucocorticoid dose of 7,620 mg/m^2^ [IQR 2,547, 12,903].

At last BIA assessment, the median age of the NCCAH group was 12.3 years [IQR 8.9, 15.4], 19 (25.3%) were overweight/obese (BMI z-score ≥1.036), among them 10 (13.3%) were obese (BMI z-score ≥1.645). The median number of hours spent per week engaging in physical activity did not differ between groups (NCCAH = 3 [IQR 1, 5] vs controls = 3 [IQR 1, 4]), *p* = 0.978). Comparative analyses of the clinical and metabolic characteristics of the NCCAH cohort and their healthy controls at last BIA assessment are presented in **Table 3**.

**Table 3:** Clinical and metabolic characteristics of NCCAH and their healthy controls at last BIA assessment.

	NCCAH	Healthy controls	*p*-value
Age, years	12.3 [8.9, 15.4]	11.5 [8.7, 14.6]	0.526
Anthropometrics			
Height, z-score	-0.01 ± 0.94	-0.36 ± 0.79	**0.004**
Weight, z-score	0.40 ± 0.99	-0.31 ± 1.00	**<0.001**
Body mass index, z-score	0.47 ± 0.97	-0.19 ± 1.04	**<0.001**
Blood pressure, percentiles			
Systolic BP	72.0 [56.0, 83.5]	73 [49.3, 85.8]	0.885
Diastolic BP	67.0 [50.0, 77.0]	60.5 [45.0, 79.0]	0.285
Body composition parameters			
Fat percentage	24.2 [20.8, 29.5]	22.7 [19.4, 25.9]	**0.041**
Truncal fat percentage	18.7 [15.55, 23.95]	17.1 [14.43, 21.23]	**0.039**
Muscle-to-fat ratio, z-score	-0.78 [-1.09, -0.11]	-0.35 [-0.94, 0.15]	**0.029**
Laboratory evaluation			
Glucose, mg/dL	83 [81,91]	85 [81, 89]	0.734
Cholesterol, mg/dL	160.6 ± 26.3	165.5 ± 26.4	0.339
LDL-c, mg/dL	87.1 [71.2, 104.7]	91.9 [80.6, 106.0]	0.190
HDL-c, mg/dL	54 [47, 64]	54 [49, 61]	0.832
Triglycerides, mg/dL	69.5 [50.3, 94.5]	67 [51, 97]	0.920
Non-HDL-c, mg/dL	101.3 [83.9, 119.8]	107 [96, 119.4]	0.213
TG : HDL-c ratio	1.26 [0.89, 1.83]	1.16 [0.94, 1.94]	0.904

Data are expressed as mean and standard deviation or median [interquartile range]. A p value of ≤0.05 was considered significant. Bold indicates significant.

BP, blood pressure; LDL-c Low density lipoprotein cholesterol; HDL-c High density lipoprotein cholesterol; TG, triglycerides

Fifteen (20%) patients with NCCAH had documented comorbid conditions, the most common of which was ADHD in 10 individuals (13.3%, of whom four were treated with stimulant medications), Hashimoto hypothyroidism in 2 (2.7%, well-controlled with levothyroxine), celiac disease in 1 (1.3%, seronegative under gluten-free diet), developmental delay in 1 (1.3%, treated with risperidone), and anxiety disorder in 1 (1.3%, treated with selective serotonin reuptake inhibitors). Fourteen patients with NCCAH had documented dietary vitamin supplementation: 11 (14.7%) received vitamin D, 2 (2.7%) received vitamin B12, and 1 (1.3%) was treated with an iron supplement. The mean (± SD) or median [IQR] serum chemistry levels in the NCCAH group were: glucose 83 mg/dL (81, 91), total cholesterol 160.6 ± 26.3 mg/dL, LDL-c 87.7 ± 22.2 mg/dL, HDL-c 56.4 ± 12.2 mg/dL, triglycerides 69.5 mg/dL [50.25, 94.5], non-HDL-c 101.3 mg/dL [83.9, 119.8], and TG : HDL ratio 1.26 [0.89, 1.83].

Age at gonadarche in the NCCAH group was similar to that of the healthy controls and age at adrenarche was significantly younger than the healthy controls [(9.8 ± 1.6 years vs. 10.1 ± 1.6 years, *p* = 0.419) and 8.6 ± 1.9 years vs. 10.3 ± 1.6 years, *p<* 0.001), respectively]. Among the adolescent girls, no differences were found in median age at menarche between the NCCAH and healthy control groups (12.8 years [IQR 12.1, 13.8] and 12.8 years [IQR 12, 13], *p* = 0.709). Post-menarcheal females (22 NCCAH patients and 27 controls) reported similar rates of regular menses in ~73% (72.7% NCCAH patients and 74.1% controls), while irregular menses were reported in 9.1% of the NCCAH patients and 22.2% of the controls. Use of oral contraceptives was reported in 18.2% of the NCCAH patients and 3.7% of the healthy controls. During the course of follow-up, 10 (4 boys) pubertal subjects with NCCAH received GnRHa therapy: 6 (2 boys) were treated with GnRHa alone for the indication of precocious puberty and 4 were treated with a combination of GnRHa and GH due to predicted short stature in adulthood. All 10 subjects were off GnRHa and GnRHa/GH treatment at the time of BIA assessment.

**Figure 2:**
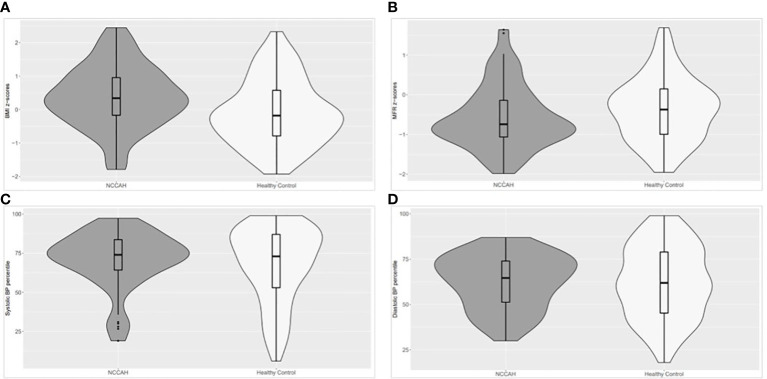
Violin plot (distributions of numeric data using probability density curves) depicting the distribution of the median of average BMI z-scores, MFR z-scores, and BP percentiles (systolic and diastolic) in the NCCAH study group and their healthy controls. NCCAH patients are presented in dark grey and healthy controls in white. The violin includes a box plot and summarizes five values: the minimum, first quartile, median, third quartile, and maximum, enabling simultaneous visualization of multiple distributions for comparison. The width describes how frequently that value occurs in the data set: wider regions of the density plot indicate that the value occurs more frequently, and narrower regions indicate that the value occurs less frequently. **(A)** The NCCAH study group had significantly higher BMI z-scores than their healthy controls (median [IQR]: 0.34 [-0.17, 0,96] and -0.18 [-0.79, 0.58], respectively, *p* = 0.001). **(B)** The NCCAH group had significantly lower MFR z-scores than their healthy controls (median [IQR]: -0.74 [-1.06, -0.14] and -0.37 [-0.99, 0.15], respectively, *p* = 0.045). **(C)** The NCCAH study group had similar systolic BP percentiles as their healthy controls (median [IQR]: 74 [64.3, 83.7] and 73 [53.0, 87.0], respectively, NS). **(D)** The NCCAH group had similar diastolic BP percentiles as their healthy controls (median [IQR]: 64.7 [51.3, 74.0] and 62 [42.3, 79.0], respectively, NS).

The body composition of the NCCAH group was characterized by higher fat and muscle mass compared to the control group. The fat mass was 10.4 kg [IQR 7.15, 16.35] vs 8.2 kg [IQR 5.8, 12.68] (*p* = 0.005), and the muscle mass was 13.3 kg [IQR 9.15, 17.05] vs 10.8 kg [IQR 7.5, 14.9] (*p*=0.017). Comparative analyses and graphical depiction of the median of average MFR z-scores, BMI z-scores, and BP percentiles (systolic and diastolic) during repeated visits of the two groups are presented in **Figures 2A–D**. The NCCAH patients were characterized by significantly higher BMI z-scores (*p* = 0.001) and lower MFR z-scores (*p =* 0.045) than their controls, without significant differences in BP percentiles. Graphic visualization of the distributions of BMI z-scores, MFR z-scores, and BP values showed that the two groups had a dissimilar center, spread, and distribution of these clinical parameters **(** **Figure 2****)**. Subgroup analysis by pubertal status revealed no significant differences in MFR z-scores in both the NCCAH group and the healthy controls.

Stepwise linear regression models were applied to evaluate explanatory variables for MFR z-scores, BP percentiles, lipid profiles, and glucose metabolism values in the NCCAH group, (**Table 4**). The final model for higher MFR z-scores (R^2^= 0.253, *p <* 0.001) included higher SEP index (*p* = 0.003), lower birthweight z-scores (*p* = 0.013), and longer duration of hydrocortisone treatment (*p* = 0.023). The final model for higher systolic BP percentile (R^2^= 0.166, *p <* 0.001) included lower MFR z-scores (*p <* 0.001). The final model for higher TG/HDL ratio (R^2^= 0.116, *p* = 0.024) included lower MFR z-scores (*p* = 0.024). No significant variables were found for glucose levels.

**Table 4:** Stepwise linear regression models in the NCCAH study group.

MFR z-scores					
				95% confidence interval	
	R^2^	SE	*p* value	lower	upper
Model	0.253		**<0.001**		
Constants	β				
SEP index	0.348	0.114	**0.003**	0.121	0.575
Birthweight z-score	-0.258	0.101	**0.013**	-0.460	-0.055
Duration of hydrocortisone treatment, *years*	0.048	0.002	**0.023**	0.001	0.007
**Systolic BP percentiles**					
				95% confidence interval	
	R^2^	SE	*p* value	lower	upper
Model	0.166		**<0.001**		
Constants	β				
MFR z-score	-9.751	2.668	**<0.001**	-15.077	-4.425
**TG : HDL-c ratios**					
				95% confidence interval	
	R^2^	SE	*p* value	lower	upper
Model	0.116		**0.024**		
Constants	β				
MFR z-score	-0.300	0.128	**0.024**	-0.558	-0.042

Stepwise linear regression models were applied to evaluate explanatory variables for MFR z-scores, systolic BP percentiles, and TG : HDL-c ratios. Variables entered into the models: sex, SEP index, family history of obesity, age (at diagnosis and at BIA), perinatal characteristics (gestational age, birthweight z-scores), and hydrocortisone exposure (mean dose, treatment duration, cumulative dose); MFR z-scores were also included for SBP percentiles and TG : HDL-c ratios. Girls compromised the reference group for sex, and lack of family history of obesity compromised the reference group. A p‐value of ≤0.05 was considered significant. Bold indicates significant.

NCCAH, non-classic congenital adrenal hyperplasia; MFR, muscle to fat ratio; SEP, Socioeconomic position; BP, blood pressure; TG, triglycerides; HDL-c High density lipoprotein cholesterol.

## Discussion

In this observational study children and adolescents with NCCAH did not have a greater rate of overweight and obesity than their healthy sex- and age-matched controls. They did, however, have an unfavorable body composition, with an imbalance between muscle and adipose tissue. Factors, such as a lower SEP and higher birthweight z-scores adversely affected their body composition while the duration of hydrocortisone therapy was found to be beneficial.

Our results revealed that youth with NCCAH had a similar prevalence of overweight/obesity compared to the general Israeli pediatric population (44). These results are in line with a recent Israeli study on the prevalence of overweight/obesity in adolescents and young adults with NCCAH (11). Most studies report on an increased BMI in children and adults with classic and non-classic CAH (8, 45–51). Of note, the focus of those studies was on the classic form of CAH, while the NCCAH form was under-represented. A Swedish study that compared patients with 21-hydroxylase deficiency who were listed in national population registries as having either salt-wasting, simple virilizing, or NCCAH found that obesity was consistently increased in all subgroups and most pronounced in patients with NCCAH (52). Although our subjects with NCCAH did not have an increased rate of obesity, they did have higher BMI z-scores on average compared to their healthy controls.

Interestingly, the body composition of our NCCAH subjects was characterized by a low MFR z-score due to higher fat mass, indicating that their muscle mass was relatively low compared to their fat mass, thus placing them at risk for sarcopenic obesity (53). Sex differences in body composition are primarily attributable to the level and action of sex steroid hormones that drive the dimorphisms during pubertal development. Sexual dimorphism in human body composition is evident from fetal life, but becomes more pronounced during puberty. At birth, males have a similar fat mass as females but are longer in stature and have greater lean mass (54, 55). Adult males having greater muscle mass (56), larger and stronger bones (57), and reduced limb fat, with a similar degree of central abdominal fat. Females have a more peripheral distribution of fat in early adulthood (58). These differences mandate a sex-adjusted interpretation of the body composition measurement. The comparison between boys and girls with NCCAH in our study revealed similar unfavorable sex- and age-adjusted body composition parameters in both sexes. There are scarce data on body composition and the role of hyperandrogenism in subjects with NCCAH. A recent study on 30 adults (5 males) with NCCAH that assessed body composition by means of BIA reported similar lean and fat mass in comparison to healthy controls (59). Another study that assessed body composition with DEXA in 12 children and adolescents with NCCAH reported higher lean body mass adjusted for sex, age, height, and pubertal status (46). Those authors concluded that the greater lean body mass and parameters of insulin resistance in children with NCCAH most likely reflect the adverse metabolic effects of prolonged postnatal androgen excess (46). Our findings on body composition are not comparable to those of previous reports since we utilized a composite score of both muscle and fat components. The interaction between circulating androgens and body composition parameters is complex: hyperandrogenism may contribute to increased muscle mass, but contrarily, it may lead to increased regional adiposity (60). While we can offer no solid explanation for our findings, we speculate that the phenotypic spectrum of subjects with NCCAH and their ability to accumulate muscle mass are also affected by androgen receptor sensitivity (61). Variable androgen receptor sensitivity may modulate the emergence of premature adrenarche in children with NCCAH and determine their clinical manifestations (61).

Glucocorticoid therapy in subjects with NCCAH is reserved and tailored for symptomatic cases of hyperandrogenism (5). Clinicians are aware of the possible adverse metabolic implications of glucocorticoid therapy (6) and the fine balance required to avoid sustained hyperandrogenism or glucocorticoid overtreatment (7). It is encouraging that prolonged glucocorticoid exposure, with substantial cumulative doses, apparently did not adversely affect the body composition of our NCCAH patients. Moreover, duration of glucocorticoid exposure was found to harbor a protective effect on the balance between muscle and fat tissue. Early diagnosis of NCCAH and initiation of glucocorticoid therapy in a timely manner has been shown to have a beneficial effect on adult height (62). Of note, the mean daily dose of hydrocortisone in our group was low (6.59 mg/m^2^) compared to that of Eyal et al.’s multicenter study conducted in Israeli patients with NCCAH (12.8 ± 4.0 mg/m^2^) (62). Our observations support the accumulating evidence that glucocorticoid therapy aimed to alleviate androgen overproduction is advantageous.

MFR was found to be an indicator for metabolic syndrome and its components in adults (63–65). We had earlier demonstrated the predictive value of MFR z-scores in assessing CVD risk factors in youth with overweight and obesity (26), and its association with hypertension in underweight/overweight children (25). The current study expands upon the clinical implications of MFR assessment in children, namely, that a lower MFR z-score is associated with both a higher systolic BP and a higher atherogenic dyslipidemia index (TG : HDL-c ratio) in young patients with NCCAH, possibly predicting future development of metabolic syndrome in their adult life (66).

Both classic and non-classic CAH was associated with established excess cardiovascular and metabolic morbidity in a large Swedish cohort (52). However, the mechanism remains unknown since glucocorticoid dose and duration as well as other clinical characteristics were not assessed (52). In addition, previous studies usually report on the corticosteroid dosage at the time of the study while the cumulative dose during the entire treatment period remains obscure. Of note, our patients with NCCAH did not have higher rates of elevated BP or dyslipidemic lipid profiles. Further prospective studies on larger cohorts are warranted to explore the link between the diagnosis of NCCAH, the level of hyperandrogenism, glucocorticoid exposure, and excess cardiovascular and metabolic morbidity.

Early onset of metabolic complications may also stem from other factors that are unrelated to NCCAH pathophysiology. Our NCCAH group was characterized by higher rates of prematurity compared to healthy controls. Preterm birth has been strongly associated with metabolic syndrome components and cardiovascular disease in adult life (67). Of note, gestational age did not emerge as an explanatory factor in the models predicting metabolic syndrome components in our patient population.

Another key factor in determining metabolic risk is adversative socioeconomic circumstances (68). In our study, a lower SEP index was identified as significant predictor for unfavorable body composition, leading us to speculate that socioeconomic circumstances may affect the risk for metabolic derangements in our patients with NCCAH throughout life. This finding should be taken into consideration in clinical management, and the appropriate social services should be consulted.

The main limitation of this study is its retrospective design which does not allow us to establish causality between exposure to hydrocortisone and metabolic outcomes. Hydrocortisone doses were calculated according to the parent-reported doses documented in the medical files, thereby lacking data on drug accountability and introducing information bias. In addition, our study did not include questionnaires for evaluating nutritional, behavioral, and psychological aspects that may affect weight status and body composition. Of note, we recently reported that health-related quality of life was not adversely affected by NCCAH among adequately treated children and adolescents (69) Although our tertiary care center serves all sectors of the Israeli population, including patients of various ethnic origins and SEP from both urban and rural areas, this study may be prone to selection bias and may not be representative of the Israeli population with NCCAH. The main strength of the present study lies in the relatively long follow-up of a NCCAH cohort attending a single tertiary medical center and which underwent a comprehensive uniform evaluation of anthropometric, body composition, BP and pubertal status measurements by the same trained medical personnel. The use of healthy controls, and the calculation of sex- and age-adjusted z-scores/percentiles allows for comparisons between subjects and more robust interpretation of the data. To the best of our knowledge, this is the first study to explore MFR z-scores and cumulative hydrocortisone doses in a pediatric population with NCCAH.

## Conclusion

In conclusion, our findings suggest that youth with NCCAH have a body composition characterized by an imbalance between muscle and fat tissue, placing them at an increased risk for early-onset cardiometabolic derangements. Implementation of BIA as a part of routine assessment may assist in the identification of cardiometabolic risk factors in youth with NCCAH, thus enabling the physician to apply interventions to those in need of risk reduction. It is reassuring that low-dose glucocorticoid therapy in pediatric patients with NCCAH aimed to alleviate androgen overproduction does not appear to adversely affect body composition.

## Data availability statement

The raw data supporting the conclusions of this article will be made available by the authors, without undue reservation.

## Ethics statement

The studies involving human participants were reviewed and approved by Tel Aviv Sourasky Medical Center IRB. Written informed consent from the participants’ legal guardian/next of kin was not required to participate in this study in accordance with the national legislation and the institutional requirements.

## Author contributions

ABS designed the study, gathered, analyzed and interpreted the data, wrote the first draft, revised the final manuscript and decided on submission. AB conceived and designed the study, analyzed and interpreted the data, contributed to the first draft and revised the manuscript incorporating contributions from coauthors, and decided on submission. AS-B conceived and designed the study, contributed to the data used in this study, reviewed and edited the manuscript, and contributed to the discussion. MY-G assisted in statistical analysis, interpreted the data of the study and contributed to the discussion. AU, ASD, AA, AO, OE and NW contributed to the data used in this study, reviewed and edited the manuscript, and contributed to the discussion. YL conceived and designed the study, analyzed and interpreted the data, critically revised the manuscript incorporating contributions from coauthors and decided on submission. All authors have read and approved the final manuscript. YL is the guarantor of this work and, as such, had full access to all the data in the study and takes responsibility for the integrity of the data and the accuracy of the data analysis. All authors contributed to the article and approved the submitted version.

## Acknowledgments

This work was performed by ABS in partial fulfilment of the MD thesis requirements of the Sackler Faculty of Medicine, Tel Aviv University, Tel Aviv, Israel. Parts of this work were presented in abstract form at ESPE 2022. The authors are grateful to the multidisciplinary team of dedicated nurses, dieticians, psychosocial workers and physicians at the Pediatric Endocrine Unit at Dana-Dwek Children’s Hospital, and to Esther Eshkol for editorial assistance.

## Conflict of interest

The authors declare that the research was conducted in the absence of any commercial or financial relationships that could be construed as a potential conflict of interest.

## Publisher’s note

All claims expressed in this article are solely those of the authors and do not necessarily represent those of their affiliated organizations, or those of the publisher, the editors and the reviewers. Any product that may be evaluated in this article, or claim that may be made by its manufacturer, is not guaranteed or endorsed by the publisher.
